# GOAnnotator: accurate protein function annotation using automatically retrieved literature

**DOI:** 10.1093/bioinformatics/btaf199

**Published:** 2025-07-15

**Authors:** Huiying Yan, Hancheng Liu, Shaojun Wang, Shanfeng Zhu

**Affiliations:** Institute of Science and Technology for Brain-Inspired Intelligence and MOE Frontiers Center for Brain Science, Fudan University, Shanghai 200433, China; Institute of Science and Technology for Brain-Inspired Intelligence and MOE Frontiers Center for Brain Science, Fudan University, Shanghai 200433, China; Institute of Science and Technology for Brain-Inspired Intelligence and MOE Frontiers Center for Brain Science, Fudan University, Shanghai 200433, China; Institute of Science and Technology for Brain-Inspired Intelligence and MOE Frontiers Center for Brain Science, Fudan University, Shanghai 200433, China; Key Laboratory of Computational Neuroscience and Brain-Inspired Intelligence (Fudan University), Ministry of Education, Shanghai, 200433, China; Shanghai Key Lab of Intelligent Information Processing and Shanghai Institute of Artificial Intelligence Algorithm, Fudan University, Shanghai, 200433, China; Zhangjiang Fudan International Innovation Center, Shanghai 201210, China

## Abstract

**Summary:**

Automated protein function prediction/annotation (AFP) is vital for understanding biological processes and advancing biomedical research. Existing text-based AFP methods including the state-of-the-art method, GORetriever, rely on expert-curated relevant literature, which is costly and time-consuming, and cover only a small portion of the proteins in UniProt. To overcome this limitation, we propose GOAnnotator, a novel framework for automated protein function annotation. It consists of two key modules: PubRetriever, a hybrid system for retrieving and re-ranking relevant literature, and GORetriever+, an enhanced module for identifying Gene Ontology (GO) terms from the retrieved texts. Extensive experiments over three benchmark datasets demonstrate that GOAnnotator delivers high-quality functional annotations, surpassing GORetriever in realistic situations by uncovering unique literature and predicting additional functions. These results highlight its great potential to streamline and enhance annotation of protein functions without relying on manual curation.

**Availability and implementation:**

The code and data are available at https://github.com/ZhuLab-Fudan/GOAnnotator.

## 1 Introduction

Proteins are vital biomolecules that perform diverse and essential functions in living organisms. Understanding their functions is essential to unravel the mechanisms of life and drive advances in disease diagnosis, treatment, and drug development. Gene Ontology (GO) (Ashburner *et al.* 2000) was developed to systematically describe protein functions. It consists of three branches: Molecular Function Ontology (MFO), Biological Process Ontology (BPO), and Cellular Component Ontology (CCO), collectively encompassing more than 50 000 terms. The leading database for proteins and their function information, UniProt (UniProt Consortium* et al*. 2025), consists of two sections: Swiss-Prot and TrEMBL. Swiss-Prot contains over 570k proteins and provides high-quality, manually curated annotations derived from expert review of literature and sequence analysis (Gasteiger *et al.* 2001). This process, while reliable, is labor-intensive and time-consuming, limiting its scalability to the rapidly growing volume of protein sequence data (Wang *et al.* 2021). In contrast, TrEMBL contains over 250M proteins, uses automated annotations to accommodate this growth but often lacks depth and accuracy (O’Donovan *et al.* 2002). Consequently, only <0.1% of proteins in UniProt have been assigned experimental functional annotations. This imbalance highlights the urgent need to develop efficient and scalable automated protein function prediction/annotation (AFP) methods.

Numerous methods have been proposed to address AFP by leveraging various data sources (Zhou *et al.* 2019), such as protein sequence (Cao and Shen 2021, Kulmanov and Hoehndorf 2020, 2022), structure (Gligorijević *et al.* 2021, Lai and Xu 2022, Boadu *et al.* 2023), protein–protein interaction (You *et al.* 2021), and protein language model(Rives *et al.* 2021), with literature-based AFP (You *et al.* 2018, Yan *et al.* 2024) being a significant subset. For instance, DeepText2GO (You *et al.* 2018) generates text-based representations for each protein using D2V and TF-IDF of relevant literature. Moreover, GOCurator, the top-performing method in the fifth Critical Assessment of Protein Function Annotation (CAFA5) (Friedberg *et al.* 2023), has been developed using ensemble approaches that combine various types of data sources, where its core component, known as GORetriever (Yan *et al.* 2024), is also a literature-based AFP. GORetriever utilizes a two-stage information retrieval framework to identify and rank GO terms based on relevant literature, excelling particularly with “difficult” test proteins that share limited sequence similarity with training proteins. The success of literature-based methods highlights the efficiency and potential of leveraging scientific literature for protein function annotation. However, these approaches heavily depend on expert-curated literature, limiting their applicability to the vast majority of uncurated TrEMBL proteins and hindering the identification of functions described in literature but not yet curated by Swiss-Prot experts. It is therefore crucial to develop a high-performance literature-based AFP method that operates independently of manual curation.

Several automatic tools have been utilized in UniProt for facilitating biomedical literature-based text mining tasks. For example, UniProt employs tools like PubTator_Tmvar (Wei *et al.* 2018) and pGenN (Ding *et al.* 2017), which specialize in specific domains such as finding human variants or plant-related literature. Among these tools, PubTator (Wei *et al.* 2024) is one of the most widely used for assisting protein function annotation. It processes literature in three stages: named entity recognition, identifier mapping, and relationship extraction, with query normalization employed to enhance accuracy. However, PubTator’s reliance on a recognition-mapping approach limits its coverage, as it fails to retrieve results when entities are not properly recognized. This underscores the need for a model that leverages deep semantic understanding to retrieve information, thereby overcoming the limitations of entity-based methods.

**Figure 1.**
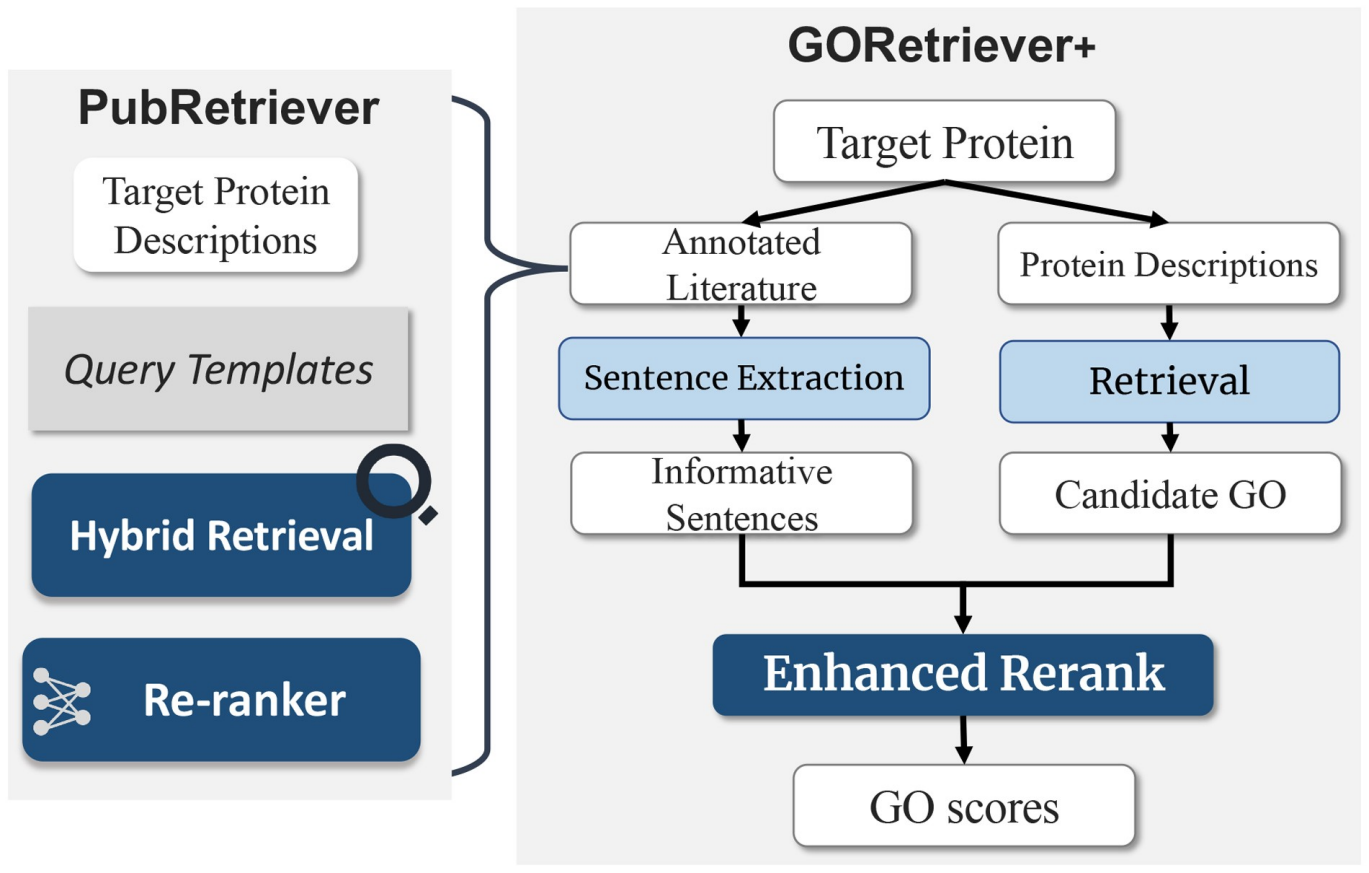
Workflow of GOAnnotator. The left part represents ‘PubRetriever’, comprising hybrid retrieval and a re-ranker designed to retrieve relevant literature effectively. The right part illustrates ‘GORetriever+’, which updates the *Rerank* component of the original GORetriever to improve GO term prediction.

To address these challenges, we have developed “GOAnnotator”, a high-performance framework designed for large-scale protein function annotation through automated literature retrieval and analysis. As shown in Fig. 1, GOAnnotator comprises two key modules: a literature search module (“PubRetriever”) and a function annotation module (“GORetriever+”). In the literature search module, PubRetriever retrieves the most relevant articles from the MEDLINE database for a given target protein. It employs a two-stage deep literature retrieval approach: first, it uses various query templates to capture multi-granularity information; then, a re-ranker identifies the most relevant articles. To enhance the re-ranker’s performance, we utilize a downstream-aligned training strategy. In the function annotation module, GORetriever+, a two-stage literature-driven protein function annotation framework, assesses the relevance between retrieved literature and candidate GO terms to generate the final GO term list. It enhances the performance of GORetriever by re-training its *Rerank* component that uses literature information to rerank candidate GO terms.

By performance comparison with GORetriever which relies on expert-curated literature, we validated the effectiveness of GOAnnotator on three benchmark datasets designed for distinct scenarios: (I) Proteins from Swiss-Prot with comprehensive functional annotations and supporting literature (used in GORetriever). (II) Proteins that first received experimental annotations in 2024 and have a relatively abundant literature. (III) Proteins with minimal prior knowledge in TrEMBL. Among these scenarios, Scenario III is the most challenging for both GORetriever and GOAnnotator due to the limited availability of expert-curated literature and relevant articles in MEDLINE, followed by Scenarios II and I. Notably, Scenarios II and III reflect more realistic situations, as the vast majority of proteins have little to no expert-curated literature. Extensive experimental results demonstrate that GOAnnotator outperforms GORetriever in Scenarios II and III, while achieving comparable performance in scenario I. Further analysis reveals that GOAnnotator overcomes limitations associated with expert curation and PubTator by retrieving distinct but functionally informative literature. Moreover, GOAnnotator is capable of identifying functions missed by other competitive methods. These findings highlight GOAnnotator’s robustness and effectiveness across diverse annotation scenarios. Collectively, these results underscore the potential of GOAnnotator to streamline and enhance protein function annotation without relying on manual curation.

## 2 Materials and methods

As shown in Fig. 1, GOAnnotator integrates two key components: PubRetriever and GORetriever+. PubRetriever retrieves relevant MEDLINE literature by combining “hybrid BM25-based sparse retrieval” with a “downstream-aligned re-ranker,” significantly improving retrieval performance. On the other hand, “GORetriever+” enhances GORetriever by re-training its *Rerank* component, enabling robust task-specific performance.

### 2.1 Preliminaries: BM25 and GORetriever

As a classical probabilistic-model-based information retrieval technique, “BM25” is a ranking function used to estimate the relevance of retrieved documents for a given query(Robertson and Jones 1976). The core idea is to compute a relevance score based on the relationship between each query term and the document, as well as their importance. Consequently, longer queries often lead to higher BM25 scores for documents.

“GORertriever” is a two-stage deep retrieval-based approach for AFP. It begins by retrieving an initial set of candidate GO terms from annotated proteins in the training data that share similar descriptions with the target protein (*Retrieval*). Simultaneously, to create effective protein representations and minimize noise, GORetriever extracts the most informative sentences from the annotated literature for each protein (*Sentence Extraction*). In the second stage, the *Rerank* component rescores the initial results, prioritizing relevant GO terms through deep semantic matching between proteins and GO terms using their textual content.

### 2.2 PubRetriever I: hybrid retrieval for multi-grained information

**Figure 2.**
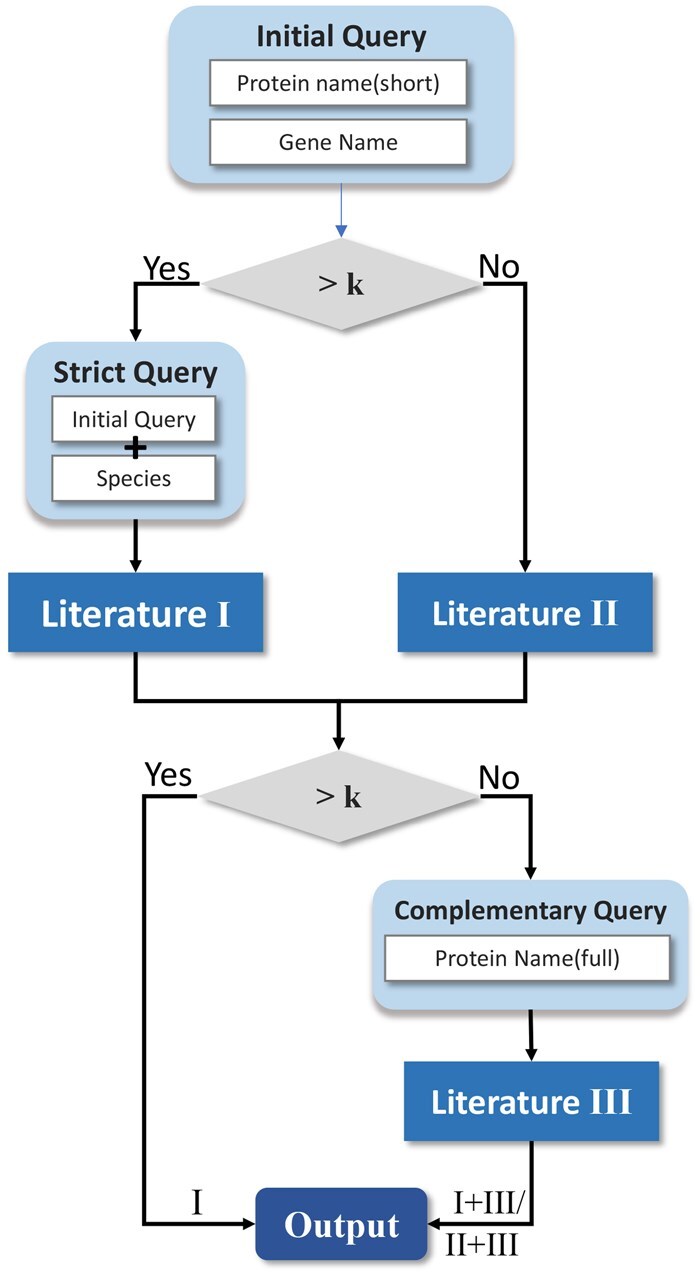
Illustration of the fine-grained retrieval.

To retrieve relevant documents, we first employ a sparse retriever (BM25, Robertson and Zaragoza 2009) using protein descriptions, including names and species. Since UniProt proteins may have multiple names (as illustrated in Table 1), a straightforward approach to retrieving relevant literature is to combine all these names into a single query, referred to as “Full-Information Retrieval” (FIR). However, longer queries can compromise precision, leading to a loss of critical information. To address this issue, we propose “Fine-Grained Retrieval” (FGR) as a complementary approach to FIR. This method uses fine-grained query templates to target specific protein information, enhancing precision, as depicted in Fig. 2.

**Table 1.** Protein descriptions of protein Q6V4H0.

Protein ID	Q6V4H0
Protein name(full)	8-hydroxygeraniol dehydrogenase
Protein name(short)	Cr10HGO
Gene name	10HGO
Species	Catharanthus roseus

We begin by extracting the short name or gene name as the *initial query* to retrieve *k* documents. Since orthologous genes across species often share similar names, relying solely on these identifiers may not uniquely identify the target protein. To refine the search, if more than *k* documents are retrieved, we use a *strict query* that combines the initial query with the species name. To reduce noise introduced by the species name, the initial query is repeated four times before concatenation, ensuring a stronger focus on the target protein. If fewer than *k* documents are retrieved, a *complementary query* using the protein’s full name supplements the results.

BM25 scores are recorded for all retrieved documents. Documents retrieved under multiple templates (initial, strict, or complementary query) are assigned an average BM25 score. The results are ranked by these scores, and the top *k* are selected. To account for BM25’s tendency to favor longer queries, scores are normalized by query length as follows:


$${\text{score}}_{\text{norm}}=\frac{\text{score}}{\text{number}\;\text{of}\;\text{words}}.$$

FGR excels at identifying documents that are highly relevant to specific proteins but may struggle with novel proteins due to inconsistent naming conventions. In contrast, FIR, while less precise, can capture documents related to homologous or functionally similar proteins, which is beneficial for function prediction. To combine the strengths of both approaches, we employ a hybrid retrieval strategy: retrieving *k* documents using FGR and another *k* using FIR. The combined results are then passed through a re-ranker to select the most relevant documents.

### 2.3 PubRetriever II: downstream-aligned training to build a better literature re-ranker for protein annotation

Algorithm 1 Downstream-aligned Re-Ranker Training

**Require:** Protein information P={p,G}, where *p* is the protein description and *G* is the annotated GO sets; document sets $D=\{D_{\text{hr}},D_{\text{sp}}\}$; downstream predictor predictor(·); trainable re-ranker f(·)

**Ensures:** Ranking loss derived from the triplet $\left(p,D^{+},D^{-}\right)$

1: ***Step I: Compute pseudo-label:***

2: Extract $g_{\text{leaf}}$ (leaf node of GO tree from *G*)

3: **for all**  $d_{i}\in D$  **do**

4: ${\hat{y}}_{i}=\text{predictor}(\text{concat}(p,d_{i}),g_{\text{leaf}})$

5: $\text{concat}(p,d_{i})=〈“\text{The}\;\text{protein}\;\text{is}”,p,“\text{The}\;\text{description}\;\text{is}”,d_{i}\rangle$

6: **end for**

7: ***Step II: Dynamically adjust thresholds:***

8: **if**  $\exists d_{j}\in D_{\text{sp}}\;\text{with}\;{\hat{y}}_{j}>0.5$  **then**

9:   $t_{\text{sp}}=0.5$, $t_{p}=0.9$, $t_{n}=0.8$

10: **else**

11: $t_{\text{sp}}=t_{p}=t_{n}=\frac{1}{\left|D_{\text{sp}}\right|}\sum_{d_{i}\in D_{\text{sp}}}{\hat{y}}_{i}$

12: **end if**

13: ***Step III: Construct training set:***

14: **for all**  $d_{i}\in D$  **do**

15:   **if**  ${\hat{y}}_{i}>t_{sp}$ and $d_{i}\in D_{\text{sp}}$  **or**  ${\hat{y}}_{i}>t_{p}$ and $d_{i}\in D_{\text{hr}}$  **then**

16:    Add $d_{i}$ to $D^{+}$

17:   **else if**  ${\hat{y}}_{i}\leq t_{n}$ and $d_{i}\in D_{\text{hr}}$  **then**

18:    Add $d_{i}$ to $D^{-}$

19:   **end if**

20:  **end for**

21: ***Step IV:*** Train re-ranker f(p,d) with ranking loss derived from the triplet for each $p\in P_{\text{train}}$:

$$\begin{array}{c} L_{\text{rank}}^{p}=\sum_{i=1}^{N}\max(0,\text{margin}-f(p,d_{i}^{+})+f(p,d_{i}^{-}))\\ d_{i}^{+}\in D^{+},d_{i}^{-}\in D^{-},N=\min(|D^{+}|,|D^{-}|)\\ f(p,d)=\sigma (W^{T}\cdot \mathrm{BER}T_{\mathrm{CLS}}(<[\mathrm{CLS}],\mathrm{prompt},[\mathrm{SEP}],d>)+b)\\ \mathrm{prompt}:¨\{\{p\}\}.\;\mathrm{The}\;\{\{\mathrm{Task}\;\mathrm{Definition}\}\}\;is¨ \end{array}$$

For a given target protein, after retrieving 2*k MEDLINE documents through hybrid retrieval (PubRetriever I), we need to train a re-ranker to optimize document relevance by maximizing alignment between protein descriptions and relevant (positive) documents while minimizing relevance with negative ones. Although expert-annotated literature in Swiss-Prot can be used as positive samples and other BM25-retrieved documents as negatives, this approach encounters several challenges: (i) Expert-annotated literature is skewed toward specific journals, potentially misclassifying relevant documents with high BM25 scores as negative. (ii) Curators rely on full-text articles to identify protein-related literature, whereas typically only titles and abstracts can be accessed by automated tools, which may lack key functional information. (iii) The functional relevance of expert-annotated literature to the target protein is often general and may not align well with the downstream task, as it is not specifically tailored to MFO, BPO, or CCO.

**Figure 3.**
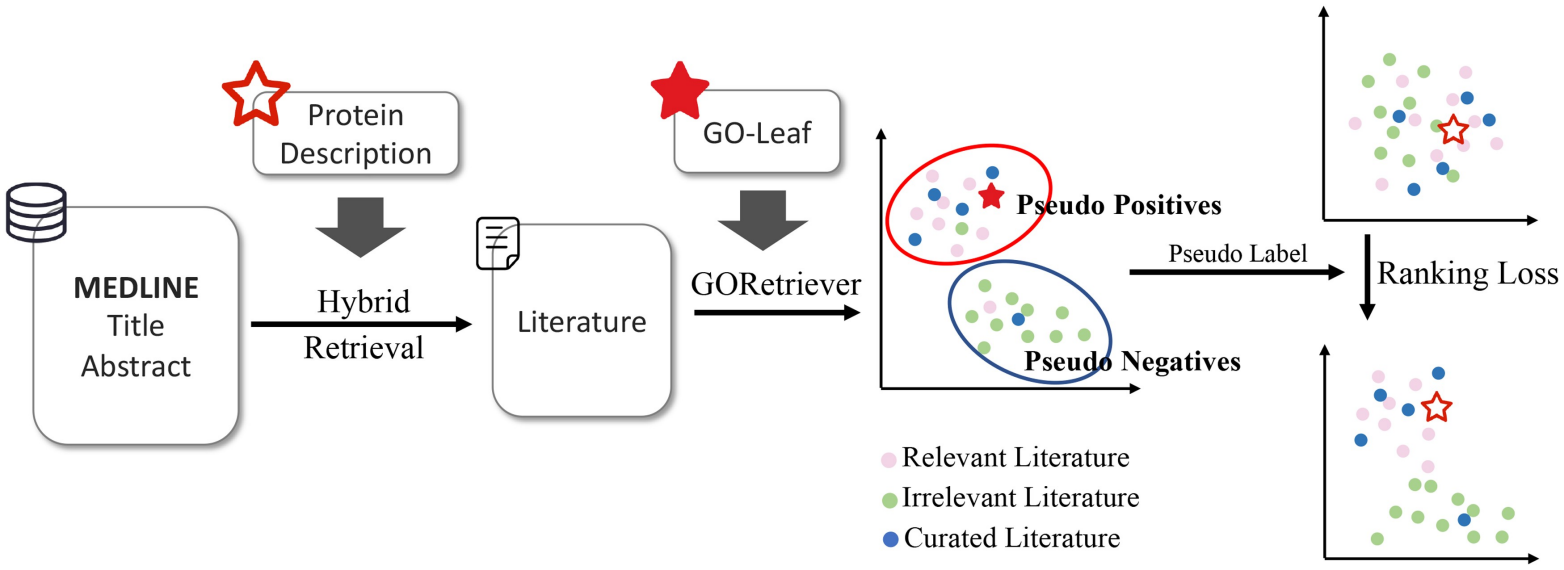
Illustration of downstream-aligned training. Relevant literature is first retrieved using hybrid retrieval based on protein descriptions. The *Rerank* component of GORetriever then evaluates the relevance of GO leaf nodes with the retrieved or curated literature. Pseudo-labels are used to divide the literature into positives and negatives, followed by applying a ranking loss to train the literature re-ranker.

To address these challenges and align the re-ranker with functional annotation, we employ a downstream predictor to generate pseudo-labels. The core idea is straightforward: only literature that contributes to downstream annotation tasks is considered relevant. As illustrated in Fig. 3 and Algorithm 1, we first use the downstream predictor to assess the relevance between functional information of target protein (GO leaf node descriptions, which serve as ground truth for downstream tasks) and unlabeled literature, comprising documents from hybrid retrieval ($D_{\text{hr}}$) and expert-curated documents in Swiss-Prot ($D_{\text{sp}}$), generating pseudo-labels. Next, we dynamically adjust thresholds based on the relevance distribution to ensure that the Swiss-Prot entries classified as positive contain sufficient information, thereby maintaining the quality of additional training pairs. Using the pseudo-labels and thresholds, we split $D=\{D_{\text{sp}},D_{\text{hr}}\}$ into $D^{+}$ and $D^{-}$ to construct the training dataset. Notably, the *Rerank* component of GORetriever (see the following subsection for the detail) is used as the downstream predictor. This serves two purposes: first, it provides stricter evaluation criteria; second, to prevent training biases from influencing the re-ranker, we use separate models for training (GORetriever) and prediction (GORetriever+). This separation ensures that downstream-aligned training does not compromise the quality of the final functional annotation.

After training, PubRetriever filters results based on relevance scores to provide the most informative literature for functional annotation tasks.

The threshold is set to 0.5, aligning with the margin of 0.1 used during training, as the relevance scores typically fall within the range of [0.4, 0.6]. To ensure efficiency, the number of filtered documents is capped at 10. If no documents meet the threshold, the highest-scoring document is selected to ensure input completeness for GORetriever+.

This process ensures alignment between the re-ranker and downstream tasks, reducing dependence on curated literature and improving performance on less-studied proteins.

### 2.4 GORetriever+: enhancing the robustness of GORetriever by re-training the rerank component

After obtaining relevant literature, GOAnnotator utilizes GORetriever+, an enhanced version of GORetriever to annotate functions based on experimental findings in textual information. Although GORetriever achieved good performance on proteins with literature annotations from Swiss-Prot, when dealing with proteins that lack sufficient expert-curated literature in Swiss-Prot, its performance was less effective. Because when training it assumes that all the input text is related to target protein, but this is not always the case in practice. To reduce the bias, we collect literature annotations from both Swiss-Prot and TrEMBL, then mix them and extract informative sentences to train a more robust and effective *Rerank* model, which constitutes GORetriever+. Further analysis shows GORetriever+ excels in complex scenarios, particularly with diverse literature sources.

## 3 Experimental setup

### 3.1 Datasets

We obtained GO annotations from Swiss-Prot, GOA (http://www.ebi.ac.uk/GOA) (Huntley *et al.* 2015), and GO (http://geneontology.org/page/download-annotations). Following the settings of CAFA5, we only used GO term annotations supported by experimental evidence codes or inferred by curators (“EXP”, “IDA”, “IPI”, “IMP”, “IGI”, “IEP”, “TAS”, “IC”, “HTP”, “HDA”, “HMP”, “HGI”, “HEP”), as well as the Gene Ontology definitions from the “go-basic.obo” file released on 1 January 2023. Following GORtrievier, we extracted protein sequences, descriptions, and PubMed identifiers (PMIDs) from UniProt, where PMIDs were used to collect expert-curated literature (title and abstract) from MEDLINE. The protein descriptions, as shown in Table 1, consist of protein names, gene names, and species information.

Same as GORetriever, our training set consists of proteins with experimental annotations and expert-curated literature prior to 2023–07. To evaluate GOAnnotator under different scenarios, we designed three benchmark datasets: (i) **GOR2023**: Simulates proteins with comprehensive functional annotations and abundant supporting literature. The dataset was originally compiled in GORetriever study for benchmarking the performance of different AFPs. It includes 1000 Swiss-Prot proteins randomly selected based on the species distribution of the CAFA5 test set, which was experimentally annotated before August 2023. (ii) **SP2024**: Represents proteins with newly added experimental annotations and relatively abundant literature. It includes all Swiss-Prot proteins that received their first experimental annotation between January 2024 and October 2024, with expert-curated literature in the December 2023 version of Swiss-Prot. This dataset evaluates GOAnnotator’s ability to annotate novel proteins based on literature (published before December 2023) uncovered by PubRetriever. (iii) **TR2024**: Focuses on proteins with minimal prior knowledge, including those present in TrEMBL until December 2023 and newly annotated with experimental functions between January 2024 and October 2024. This dataset tests GOAnnotator’s ability to predict functions for poorly characterized proteins with incomplete annotations. Note that GOR2023 and TR2024 are more realistic, as the vast majority of proteins do not have expert-curated literature. The statistics of these datasets are shown in Table 2.

**Table 2.** Number of proteins across GO domains in training and test sets.^a^

	MFO	BPO	CCO
**Train**	79 866	90 014	95 988
**GOR2023**	882	861	811
**SP2024**	178	369	368
**TR2024**	222	722	565

a The species distribution is shown in the Supplementary material.

### 3.2 Evaluation metric

We evaluate GOAnnotator’s performance using the CAFA5 evaluation metric, the weighted maximum F-measure (wFmax), which balances precision and recall while accounting for the varying importance of different GO terms (Friedberg *et al.* 2023). The wFmax is computed by maximizing the F-measure over different thresholds τ, as detailed in the Supplementary Material.

We also evaluate prediction performance using additional metrics ($S_{\min}$), as detailed in the Supplementary Material. The conclusions drawn from $S_{\min}$ are consistent with those based on $F_{\max}$.

### 3.3 Competing methods

Since we focus on text-based AFP, the most related method is GORetriever. To fully explore the role of GOAnnotator in AFP, we also include some other sequence-based, network-based, text-based, and protein language model-based methods used in GORetriever in comparisons. These methods include all important components in NetGO3.0 (BLAST-KNN, Net-KNN, LR-InterPro, LR-ESM, LR-Text) (Wang *et al.* 2023), DeepGOCNN and DeepGOPlus (Kulmanov and Hoehndorf 2020), SVM-ESM2 (a variant of LR-ESM with better performance using ESM2), LR-ProtST (a sequence-based method but using both sequence and protein description for embedding), and GORetriever. We also evaluate a variant of GOAnnotator, GOAnnotator_PubTator, which uses PubTator to retrieve relevant literature, with a maximum of 10 documents retrieved per protein. More details are provided in the Supplementary Material.

### 3.4 Experimental settings of PubRetriever

Because the test set consists of proteins annotated after 2024, we extract the title and abstract of documents indexed in the 2023 version of MEDLINE (https://ftp.ncbi.nlm.nih.gov/pubmed/baseline/) and remove the documents without ISSN identifier or abstract entry to assure the quality. We set *k* as 50 in hybrid retrieval. To simplify downstream-aligned training, we collect proteins from Train dataset with a single leaf node in the annotated GO tree. To train the re-ranker, we set the batch size as 8 and the warm-up ratio as 0.1 using BioLinkBERT(Yasunaga *et al.* 2022) as a Cross-Encoder (Nogueira *et al.* 2020).

## 4 Experimental results

### 4.1 GOR2023: GOAnnotator achieves accurate protein functional annotation comparable to GORetriever

In Table 3, the left part shows the results of GOAnnotator and competing methods on GOR2023, while the right part reports the performance of all prediction methods on GOR2023-PT. GOR2023-PT refers to the subset of GOR2023 proteins whose documents were retrieved using PubTator that relies on named entity recognition and identifier mapping.

**Table 3:** . Performance comparison of competing methods on the GOR2023 dataset.^a^

	GOR2023				GOR2023-PT			
Method	MFO	BPO	CCO	Ave. wFmax	MFO	BPO	CCO	Ave. wFmax
	(882)	(861)	(811)		(503)	(493)	(436)	
	Sequence-based method							
BLAST-KNN^b^	0.644	0.471	0.595	0.570	0.628	0.437	0.610	0.558
LR-InterPro^b^	0.627	0.465	0.591	0.561	0.642	0.440	0.621	0.568
LR-ESM^b^	0.632	0.449	0.607	0.563	0.637	0.422	0.616	0.558
SVM-ESM2	0.658	0.462	0.622	0.581	**0.656**	0.437	0.625	0.573
LR-ProtST^b^	0.649	0.462	0.625	0.579	0.646	0.433	**0.633**	0.571
DeepGOCNN	0.573	0.409	0.547	0.510	0.361	0.273	0.461	0.464
DeepGOPlus	0.645	0.478	0.604	0.576	0.639	0.455	0.625	0.573
	Network-based method							
Net-KNN^b^	0.400	0.458	0.596	0.485	0.409	0.440	0.599	0.483
	Text-based method							
LR-Text^b^	0.520	0.486	0.619	0.541	0.529	0.467	0.626	0.541
GORetriever^b^	**0.659**	**0.545**	**0.653**	**0.619**	0.651	**0.488**	0.626	**0.588**
GOAnnotator	0.651	0.532	0.641	0.608	0.646	0.481	0.626	0.584
GOAnnotator_PubTator	0.466	0.347	0.432	0.415	0.652	0.478	0.618	0.582

a The highest values are bolded and the second highest values are underlined. Numbers in parentheses denote the number of test proteins.

b Results are sourced from the original GORetriever study (Yan *et al.* 2024).

We have three main findings: (i) GOAnnotator achieves the second-best performance on both GOR2023 and GOR2023-PT, which is only slightly worse than GORetriever using expert-curated literature. For example, GOAnnotaor and GORetriever achieve an average wFmax of 0.608 and 0.619 on GOR2023, respectively, which are followed by SVM-ESM2 (0.581). (ii) GOAnnotator outperforms GAnnotator_PubTator in both protein coverage and annotation performance. Specifically, compared with GOAnnotator_PubTator, GOAnnotator achieves a higher score on GOR2023-PT (0.588 versus 0.582), demonstrating its effectiveness in identifying relevant literature through a two-stage retrieval process without relying on entity recognition or identifier mapping. Moreover, the performance gap becomes much wider on GOR2023 (0.608 versus 0.415), because PubTator covers only about 55% of all proteins, limiting its effectiveness. (iii) Literature-based methods excel in BPO and CCO, while protein language-based methods perform well on MFO. Specifically, for MFO, SVM-ESM2 achieves the best wFmax on GOR2023-PT and the second best on GOR2023. All these results highlight GOAnnotator’s effectiveness in identifying informative literature for accurate protein functional annotation, without relying on expert curation or PubTator.

### 4.2 SP2024: GOAnnotator effectively annotates novel proteins in Swiss-Prot

As shown in Table 4, we evaluate the performance of different AFP methods on SP2024. The top-performing methods on GOR2023 (GOAnnotator, GORetriever, SVM-ESM2, LR-ProtST, and DeepGOPlus), GOAnnotator’s variant (GAnnotator_PubTator), and the most well-known baseline (BLAST-KNN) are used in comparison. We have three main findings: (i) GOAnnotator achieves the best overall performance and also two branches of GO ontology, BPO, and CCO, which outperforms GORetriever using expert-curated literature. Specifically, GOAnnotator achieves the highest average wFmax of 0.503, while GORetriever, using Swiss-Prot annotations as of December 2023, shows a lower performance of 0.496. This highlights GORetriever’s dependence on high-quality literature annotations, as its performance significantly drops for proteins with limited literature support. (ii) GOAnnotator outperforms GOAnnotator_PubTator significantly in annotating novel proteins. GOAnnotator_PubTator achieves a wFmax of 0.387, reflecting its limitations in recognizing and mapping novel proteins due to its reliance on predefined mappings. Furthermore, we present the results of different methods on SP2024-PT, a subset of SP2024 containing proteins covered by PubTator, in the Supplementary Material. The results further underscore the limitations of PubTator. (iii) Compared with the performance on GOR2023, all AFP methods show a notable decline, especially on BPO and CCO. For example, for BPO, GOAnnotator achieves a wFmax of 0.532 on GOR2023, while it achieves a wFmax of only 0.335 on SP2024. It may be because functional annotations of these novel proteins are incomplete. Some false-positive predictions by GOAnnotator may actually be correct. In the Supplementary Material, we examine the performance of GOAnnotator and competing methods on a larger dataset, SP2025, and the results are consistent with our findings.

**Table 4:** . Performance comparison of competing methods on SP2024^a^.

Method	MFO	BPO	CCO	Ave. wFmax
	(178)	(319)	(368)	
LR-ProtST	0.617	0.301	0.548	0.489
DeepGOPlus	0.644	0.306	0.527	0.492
BLAST-KNN	0.636	0.271	0.494	0.467
SVM-ESM2	**0.649**	0.302	**0.554**	0.502
GORetriever	0.624	0.327	0.537	0.496
GOAnnotator	0.620	**0.335**	**0.554**	**0.503**
GOAnnotator_PubTator	0.472	0.253	0.435	0.387

a The highest values are bolded and the second highest values are underlined. Numbers in parentheses denote the number of test proteins.

### 4.3 TR2024: GOAnnotator effectively annotates TrEMBL proteins by integrating seamlessly with other methods

As shown in Table 5, we evaluate the performance of GOAnnotator and the competing methods on the TR2024 and TR2024-SP datasets. TR2024-SP refers to a subset of TR2024 where proteins are newly added to Swiss-Prot from TrEMBL in 2024. GOAnnotator_PubTator is omitted as it is very difficult for PubTator to retrieve literature for TrEMBL proteins. Note that all these TR2024 proteins are placed in TrEMBL by the end of 2023, which means that these proteins are less studied in the literature. It is thus very challenging to annotate the function of these proteins through text-based methods.

**Table 5.** Performance comparison of competing methods on TR2024.^a^

	TR2024				TR2024-SP			
Method	MFO	BPO	CCO	Ave. wFmax	MFO	BPO	CCO	Ave. wFmax
	(222)	(722)	(565)		(53)	(68)	(56)	
LR-ProtST	0.595	0.296	0.425	0.439	0.619	0.426	0.661	0.569
DeepGOPlus	0.584	0.288	0.473	0.448	0.609	0.467	0.585	0.554
BLAST-KNN	0.587	0.286^b^	0.442^b^	0.438^b^	0.586	0.477^b^	0.591^b^	0.551^b^
SVM-ESM2	0.586	0.300	0.426	0.437	0.577	0.467	0.622	0.555
GORetriever^c^	0.296	0.221	0.424	0.314	0.294	0.210	0.427	0.310
GOAnnotator	0.319	0.253	0.494	0.355	0.369	0.380	0.536	0.428
GOAnnotator_ESM	0.507	0.305	0.451	0.421	0.619	**0.503**	**0.673**	**0.598**
GOAnnotator_BLAST	0.515	0.283^b^	0.449^b^	0.416^b^	**0.635**	0.513 ^b^	0.684 ^b^	0.611 ^b^
Consensus	0.595	**0.324**	**0.506**	**0.475**	0.614	0.496	0.644	0.585
Consensus w/o GOAnnotator	**0.614**	0.310	0.463	0.462	0.632	0.482	0.619	0.578

a The highest values are bolded and the second highest values are underlined. Numbers in parentheses denote the number of test proteins.

b Since prediction of BLAST relies on homologous proteins, it could not predict all the proteins in BPO and CCO.

c Literature comes from TrEMBL annotation.

We have three main findings: (i) GOAnnotator outperforms GORetriever on both datasets, demonstrating its ability to uncover higher-quality literature compared to UniProt’s automated tools. For instance, GOAnnotator achieves an average wFmax of 0.428 on TR2024-SP, while GORetriever achieves only 0.310. (ii) GOAnnotator underperforms SVM-ESM2 and BLAST-KNN in both datasets. For example, GOAnnotator achieves an average wFmax of 0.355 on TR2024, while BLAST-KNN and SVM-ESM2 achieve 0.438 and 0.437, respectively. This may be because GOAnnotator relies on proteins with similar descriptions to construct GO term candidates, which limits its effectiveness for TrEMBL proteins. (iii) The performance of AFP methods has improved significantly when transitioning from TR2024 to TR2024-SP proteins. For example, SVM-ESM2 achieves an average wFmax of 0.437 on TR2024 and 0.555 on TR2024-SP. Similarly, GOAnnotator achieves an average wFmax of 0.355 and 0.428 on TR2024 and TR2024-SP, respectively. This indicates that Swiss-Prot proteins have been annotated more extensively and got more attention in the literature. The only exception is that GORetriever achieves similar performance on TR2024 (0.314) and TR2024-SP (0.310), due to its reliance on TrEMBL automatic literature annotations as of December 2023, which are of limited quality.

To address the limitations in GO candidate retrieval of GOAnnotator, we experiment with various integration methods. Specifically, we develop GOAnnotator_ESM and GOAnnotator_BLAST, which replace the candidate GO terms with the top-100 predictions from SVM-ESM2 and BLAST-KNN, respectively. Results on TR2024 show that these replacement methods significantly improve GOAnnotator’s performance, achieving the best results in CCO. For proteins with comprehensive functional annotations (TR2024-SP), GOAnnotator_ESM and GOAnnotator_BLAST outperform SVM-ESM2 and BLAST-KNN, respectively. This highlights the high quality of literature retrieved by PubRetriever. These results demonstrate that the performance of GOAnnotator can be further optimized by enhancing GO candidate retrieval with sequence-based methods.

Furthermore, we employ a consensus technique to integrate predictions from GOAnnotator and competing methods. The ensemble score S(x) is calculated as:


$$S(x)=1-\prod_{i=1}^{n}\left(1-\alpha f_{i}(x)\right),$$

where $f_{i}(x)$ represents prediction scores from individual methods, and α is set to 1. This approach consistently outperforms individual methods, achieving the best overall performance on TR2024, particularly in BPO and CCO. The declining consensus score observed without GOAnnotator confirms its essential role in the ensemble method. The overlap analysis in the supplement further demonstrates that GOAnnotator can provide unique GO annotations, making it a valuable component in integrated methods.

## 5 Analysis

### 5.1 GOAnnotator predicts additional functions using unique literature

**Figure 4.**
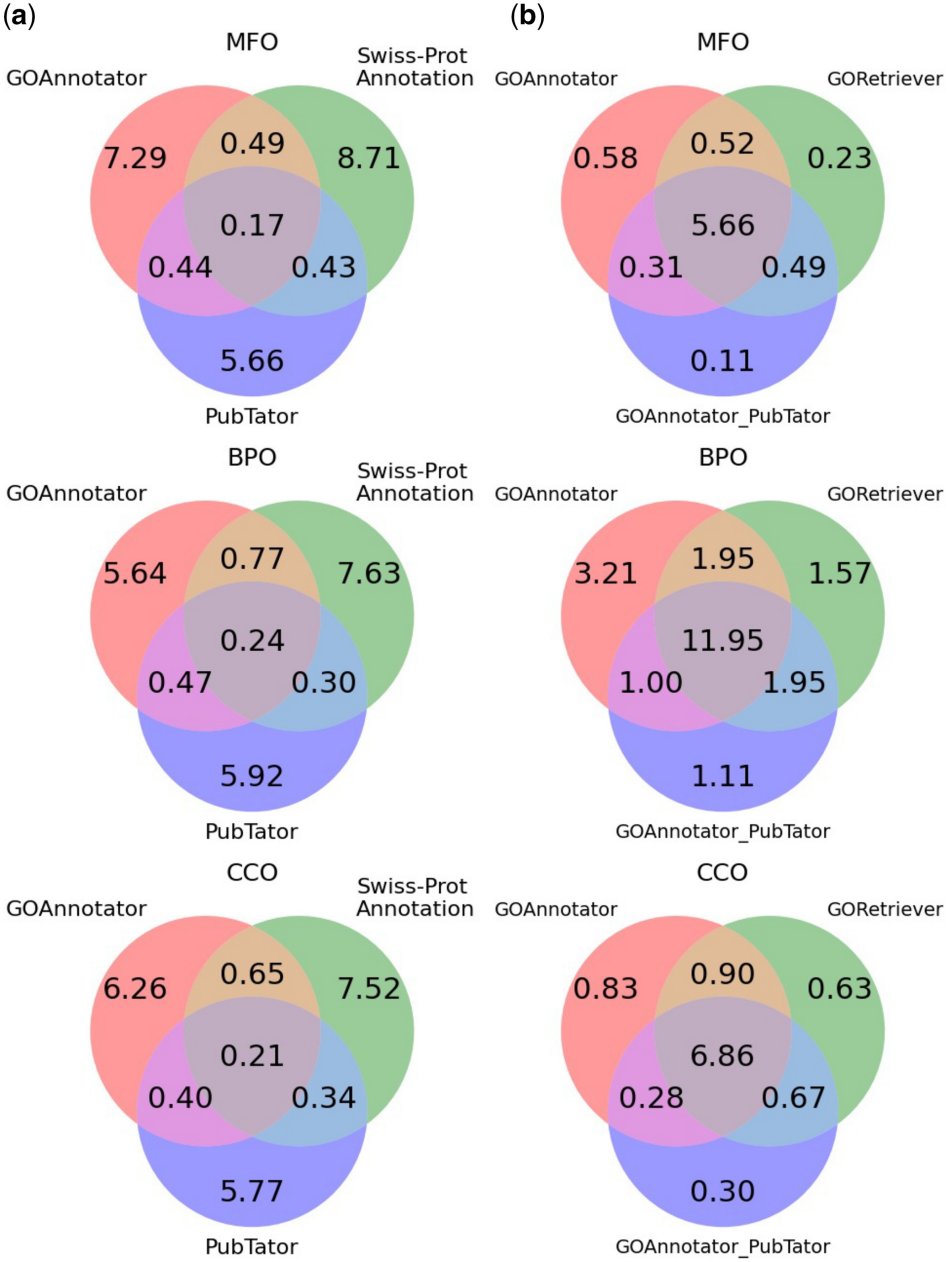
Overlap and difference of annotated literature and predicted true positive GO terms. (a) Average number of annotated/retrieved literature per protein in three methods. (b) Average number of predicted true positive GO terms per protein in three methods.

To further illustrate how GOAnnotator functions, we draw Venn diagrams in Fig. 4 to analyze the overlaps and differences among prediction results of GOAnnotator, GORetriever, and GOAnnotator_PubTator on the GOR2023-PT dataset, which is comprehensively annotated. Figure 4a shows the analysis on annotated literature. We have two main findings. (i) The overlap in retrieved/annotated literature among all three methods is small, averaging 0.19 per protein for MFO, 0.27 for BPO, and 0.24 for CCO, respectively. (ii) Compared to PubTator, GOAnnotator shows greater overlap with Swiss-Prot literature annotations, indicating that GOAnnotator retrieves more functionally relevant documents rather than only protein(gene) name matching. Figure 4b presents the overlap analysis of true positive GO terms per protein. The findings reveal that (i) GOAnnotator_PubTator predicts fewer GO terms due to its reliance on entity matching, which often results in incomplete coverage of relevant literature. Moreover, it cannot ensure that the retrieved documents specifically pertain to MFO, BPO, or CCO, causing information loss and reduced prediction accuracy; (ii) GOAnnotator exhibits a higher overlap with GORetriever than GOAnnotator_PubTator, which can be attributed to its ability to retrieve literature more similar to expert-curated literature in Swiss-Prot.

Combining insights from both figures, it is evident that GOAnnotator identifies distinct literature compared to other methods while still accurately annotating functions. This demonstrates that GOAnnotator can independently perform functional annotation using automated retrieved literature, without relying on Swiss-Prot curation or PubTator retrieval.

### 5.2 Quality evaluation of retrieved literature

To directly evaluate the quality of retrieved literature, we assessed its relevance using LLMs (Large Language Models) following Faggioli *et al.* (2023). Specifically, we randomly selected 150 proteins from GOR2023_PT (50 for each GO domain). For each protein, we extracted the definitions of its leaf GO nodes and informative sentences generated by the *Sentence Extraction* component of GORetriever+. Curators were tasked with determining whether the extracted sentences related to molecular function/biological process/cellular component or the annotated functions. The scoring criteria are as follows: 0 (Not Relevant), 1 (Partially Support), and 2 (Support), reflecting the degree of relevance and evidence provided by the literature. The detailed prompt is provided in the Supplementary Material.

To ensure a comprehensive evaluation, we employed three LLMs as automatic curators: GPT-4o (OpenAI *et al.* 2024), DeepSeek-V3 (DeepSeek-AI *et al.* 2024), DeepSeek-R1 (DeepSeek-AI *et al.* 2025), and Qwen-max (Qwen *et al.* 2025). The results, summarized in Table 6, show that (i) overall, the quality of PubRetriever-retrieved literature is higher than that of Swiss-Prot curated literature (1.59 versus 1.24 on average). The superiority of PubRetriever is especially significant in CCO (1.66 versus 0.93). (ii) For MFO, Swiss-Prot curation performs slightly better (1.63 versus 1.52 on average), possibly because expert-curated literature in Swiss-Prot emphasizes more on molecular function, while PubRetriever employs different prompts for different domains, potentially diluting its specificity for MFO. Note that these results are also consistent with the performance of GOAnnotator and GORetriever on SP2024 with a relatively abundant literature. Specifically, GOAnnotator achieves a wFmax of 0.624, 0.327, and 0.537 on MFO, BPO, and CCO, respectively. In contrast, GORetriever achieves a wFmax of 0.624, 0.327, and 0.537 on MFO, BPO, and CCO, respectively. All these results demonstrate that without expert curation, GOAnnotator can deliver high-performance AFP through automated high-quality literature retrieval.

**Table 6.** Evaluation results of LLMs.^a^

	GPT-4o	DeepSeek-V3	Qwen-max	DeepSeek-R1	Avg.
PubRetriever-retrieved literature					
MFO	1.42	1.62	1.52	1.80	1.59
BPO	1.62	1.50	1.64	1.78	1.64
CCO	1.64	1.58	1.76	1.94	1.73
Avg.	1.56	1.57	1.64	1.84	1.65
Swiss-Prot-curated literature					
MFO	1.50	1.74	1.64	1.60	1.62
BPO	1.10	1.22	1.18	1.26	1.19
CCO	0.96	0.84	1.00	1.08	0.97
Avg.	1.19	1.27	1.27	1.31	1.26

a The number is the average score on each domain.

### 5.3 Ablation study

Our ablation experiments, summarized in Table 7, evaluate the contribution of each module in GOAnnotator using the GOR2023-PT and SP2024 datasets, including well- and less-studied proteins, respectively. The results show that (i) FIR excels on less-studied proteins. FIR performs better on SP2024 (0.495 versus 0.490) when only sparse retrieval is used, as it considers more comprehensive information, yielding more diverse top-ranked results. (ii) FGR performs better on well-studied proteins. FGR achieves higher performance on GOR2023-PT (0.572 versus 0.569), as proteins in this dataset have more literature support, enabling FGR to identify more precise matches. (iii) Re-ranker enhances robustness. Re-ranker narrows the performance gap between FIR and FGR across all datasets, demonstrating its ability to improve robustness. The largest gap is observed on GOR2023-PT (0.578 versus 0.583), where FGR provides more precise results, and the re-ranker enhances contextual understanding. (iv) Hybrid retrieval with re-ranker delivers best performance. GOAnnotator achieves the best performance across all datasets when hybrid retrieval (combining FIR and FGR) is used alongside re-ranker, highlighting the effectiveness of PubRetriever.

**Table 7.** Ablation study on individual components of PubRetriever on GOR2023-PT and SP2024.^a^^,^^b^^,^^c^

Method	MFO	BPO	CCO	Ave. wFmax
GOR2023-PT				
GOAnnotator	0.646	**0.481**	0.626	**0.584**
w/o FGR	**0.647**	0.470	0.619	0.578
w/o FIR	0.642	0.472	**0.635**	0.583
w/o re-ranker				
FIR	0.635	0.465	0.607	0.569
FGR	0.643	0.457	0.615	0.572
SP2024				
GOAnnotator	0.620	**0.335**	**0.554**	**0.503**
w/o FGR	0.624	0.330	0.547	0.500
w/o FIR	0.619	0.333	0.550	0.501
w/o re-ranker				
FIR	**0.631**	0.310	0.544	0.495
FGR	0.629	0.306	0.535	0.490

a The highest values are bolded and the second highest values are underlined.

b Hybrid retrieval was not tested independently as it cannot generate a mixed and ranked list.

c FIR: Full-Information Retrieval. FGR: Fine-Grained Retrieval.

### 5.4 Case study

To further understand the capabilities of GOAnnotator, we analyze the extracted informative sentences by GOAnnotator and GORetriever. Here, we select two proteins from TR2024-SP: PbAQP (UniProt ID: A0A509APT1) and gamma-tubulin (UniProt ID: A0A644F0Y) and examine their corresponding GO terms. The former was successfully predicted by both GOAnnotator and GORetriever, while the latter was only predicted by GOAnnotator. As shown in Supplementary Table S9, both methods identify relevant sentences related to the proteins. For PbAQP, the relevant sentences of both methods discuss the plasma membrane, enabling successful annotation of the associated GO term. In contrast, for the gamma-tubulin, the relevant sentence from GORetriever refers to “perikinetosomal areas of the ventral”, which is a region functionally similar to the centrosome in certain organisms (e.g. the protozoan Giardia), making it challenging for automatic methods to identify. However, GOAnnotator successfully mines the explicit reference to “at the centrosome” due to its downstream-aligned training approach. These results highlight GOAnnotator’s ability to annotate functions for novel proteins through automated literature retrieval, effectively addressing the limitations of automated literature annotation in UniProt. Moreover, GOAnnotator sometimes outperforms GORetriever using expert-curated literature, due to its specialized retrieval and training strategy.

## 6 Conclusion

We have presented GOAnnotator, a framework for accurate protein function annotation using automated literature retrieval. For a target protein, GOAnnotator first retrieves relevant literature using hybrid retrieval and then refines the results with a downstream-aligned re-ranker. Once relevant literature is identified, GOAnnotator computes relevance scores between GO candidates and extracted informative sentences using GORetriever+, an enhanced version of GORetriever. Experiments conducted on well-studied proteins (GOR2023), newly added proteins with comprehensive functional annotations (SP2024), and proteins with developing annotations (TR2024) demonstrate that GOAnnotator achieves accurate predictions through automated literature retrieval, eliminating the need for expert curation. Further analysis shows that although the textual information used by GOAnnotator has limited overlap with Swiss-Prot annotations, it effectively predicts distinct functions and adapts to diverse scenarios.

## Supplementary Material

btaf199_Supplementary_Data

## Data Availability

The data is available at https://github.com/ZhuLab-Fudan/GOAnnotator/tree/main/data.
