# Supplementary material for: GOAnnotator: accurate protein function annotation using automatically retrieved literature
Source: Bioinformatics. 2025 Jul 15;41(Suppl 1):i410–9. doi: 10.1093/bioinformatics/btaf199 (PMC12261426; doi:10.1093/bioinformatics/btaf199)
Supplement: btaf199_Supplementary_Data [file btaf199_supplementary_data.pdf]

Supplementary Materials of "GOAnnotator: accurate protein function annotation using automatically retrieved literature"

Huiying Yan[1], Hancheng Liu [1], Shaojun Wang [1], Shanfeng Zhu [1,2,3,4,*]

[1] Institute of Science and Technology for Brain-Inspired Intelligence, Fudan University, Shanghai, 200433, China

[2] Key Laboratory of Computational Neuroscience and Brain-Inspired Intelligence (Fudan University), Ministry of Education, Shanghai 200433, China

[3] Shanghai Key Lab of Intelligent Information Processing and Shanghai Institute of Artificial Intelligence Algorithm, Fudan University, Shanghai 200433, China

[4] Zhangjiang Fudan International Innovation Center, Shanghai 200433, China

1 Details of BM25 Algorithm

The core idea of BM25 is to compute a relevance score based on the relationship between each query term and the document, as well as their importance.

The relationship between each query term (q_i) and the document (D) is measured by the term's frequency in the document. The more frequently a term appears, the higher its relevance. In BM25, this relationship is adjusted using the following formula:

$$R(q_i, D) = \frac{f(q_i, D) \cdot (k_1 + 1)}{f(q_i, D) + k_1 \cdot \left(1 - b + b \cdot \frac{|D|}{\text{avgdl}}\right)}$$

where $f(q_i, D)$ is the frequency of q_i in document D, and k_1, b are parameters that control the effect of document length.

The importance of a query term is measured using the inverse document frequency (IDF):

$$\text{IDF}(q_i) = \log \left(\frac{N - n(q_i) + 0.5}{n(q_i) + 0.5} \right)$$

where $n(q_i)$ is the number of documents containing the term q_i, and N is the total number of documents.

The final relevance score between a query Q and a document D is computed as:

$$\text{BM25}(Q, D) = \sum_{i=1}^{n} \text{IDF}(q_i) \cdot R(q_i, D)$$

2 Details of GORetriever

GORetriever is a two-stage deep retrieval-based approach for AFP, consisting of three key components: Retrieval, Sentence Extraction, and Rerank. **Retrieval** selects candidate GO terms based on similar protein descriptions. Given a target protein, *Retrieval* retrieves proteins with annotated functions based on

[1]Corresponding author: zhusf@fudan.edu.cn

their textual similarity using BM25. **Sentence Extraction** refines protein representations by identifying informative sentences from annotated literature. It employs the MonoT5 model to re-rank sentences in literature based on their similarity to protein description and then selects the Top-50% as the informative sentences. Finally, **Rerank** enhances relevance by re-scoring GO terms through deep semantic matching. It applies a Cross-Encoder predictor to re-rank retrieved GO functions based on textual similarity between informative sentences and GO description.

3 Details of Competing Methods

BLAST-KNN uses BLAST to find proteins similar to the target and assigns GO terms by evaluating the quality of sequence alignments. The prediction score reflects the contribution of each similar protein, weighted by its alignment similarity, with a strict E-value threshold of 0.001 applied in our experiments.

DeepGOPlus combines sequence similarity from the Diamond alignment tool with **DeepGOCNN**, a deep convolutional neural network model, to predict Gene Ontology (GO) terms solely from protein sequences.

LR-InterPro constructs a binary feature vector for each protein using protein families, domains, and motifs. A logistic regression classifier is then used for GO term prediction.

LR-ESM uses protein embeddings generated by ESM-1B and applies LR classifiers to predict GO terms.

SVM-ESM2 uses protein embeddings generated by ESM-2 and applies Support Vector Machine (SVM) classifiers to predict GO terms.

Net-KNN leverages protein-protein interaction (PPI) networks, such as STRING, to predict GO terms. The method identifies similar proteins within the network and calculates prediction scores by considering the relative importance of these connections based on network weights.

LR-Text applies logistic regression to predict GO terms based on text features extracted from protein annotations.

LR-ProtST incorporates both protein sequence and description information to generate embeddings, followed by logistic regression classifiers for GO term prediction.

GORetriever is a two-stage retrieval framework where the first stage retrieves candidate GO terms based on protein similarity, and the second stage re-ranks them using deep semantic matching of protein and GO term textual information.

4 Details of Evaluation Metric

The wFmax is computed by maximizing the F-measure over different thresholds τ, more details is shown in Supplemtent. as shown below:

$$\mathrm{wF}_{\max} = \max_{\tau} \left\{ \frac{2 \cdot \mathrm{wpr}(\tau) \cdot \mathrm{wrc}(\tau)}{\mathrm{wpr}(\tau) + \mathrm{wrc}(\tau)} \right\}$$

The weighted precision ($\mathrm{wpr}(\tau)$) and weighted recall ($\mathrm{wrc}(\tau)$) at threshold τ are key metrics. These are calculated as:

$$\mathrm{wpr}(\tau) = \frac{1}{m(\tau)} \sum_{i=1}^{m(\tau)} \frac{\sum_v \mathrm{IC}(v) \cdot \mathbf{1}(S(v,p_i) \geq \tau) \cdot I(v,p_i)}{\sum_v \mathrm{IC}(v) \cdot \mathbf{1}(S(v,p_i) \geq \tau)}$$

$$\mathrm{wrc}(\tau) = \frac{1}{n_e} \sum_{i=1}^{n_e} \frac{\sum_v \mathrm{IC}(v) \cdot \mathbf{1}(S(v,p_i) \geq \tau) \cdot I(v,p_i)}{\sum_v \mathrm{IC}(v) \cdot I(v,p_i)}$$

Here, $m(\tau)$ is the number of proteins with scores above threshold τ for at least one GO term, and n_e is the total number of test proteins. $S(v,p_i)$ is the predicted score for protein p_i for GO term v, and $I(v,p_i)$ indicates whether protein p_i is annotated with term v. Both precision and recall are weighted by

the information content (IC) of each term, which reflects the specificity of a GO term within the ontology [1]. The IC is defined as:

$$\mathrm{IC}(v) = \log_2 \frac{1}{\Pr(v \mid \mathcal{P}a(v))}$$

where $\mathcal{P}a(v)$ is the set of ancestor terms for v, and $\Pr(v \mid \mathcal{P}a(v))$ is the conditional probability of term v given its ancestors. This weighting ensures that more specific, informative terms are given higher importance in the evaluation.

5 Species distribution of training dataset and GOR2023

Following GORetriever, we randomly select 1,000 Swiss-Prot proteins as GOR2023 based on the species distribution of CAFA5 test superset, the species distributions of training dataset and GOR2023 are shown in Table S1, S2.

Table S1: The number of proteins of different species for training.

	MFO	BPO	CCO
**HUMAN (Homo sapiens)**	16237	11759	22783
**MOUSE (Mus musculus)**	9560	11045	10361
**DROME (Drosophila melanogaster)**	7134	10985	9154
**ARATH (Arabidopsis thaliana)**	8444	8756	8756
**RAT (Rattus norvegicus)**	5932	7000	5737
**All species (not only the above)**	79866	90014	95988

Table S2: The number of proteins of different species for GOR2023.

	MFO	BPO	CCO
**HUMAN (Homo sapiens)**	139	142	118
**MOUSE (Mus musculus)**	111	113	109
**DROME (Drosophila melanogaster)**	18	16	14
**ARATH (Arabidopsis thaliana)**	101	103	100
**RAT (Rattus norvegicus)**	47	43	50
**All species (not only the above)**	882	861	811

6 Performance Comparisons in terms of S_{min}

We also use $S_{\min}$ to evaluate the prediction performance. The *remaining uncertainty* (ru), *misinformation* (mi), and the resulting *minimum semantic distance* ($S_{\min}$) are defined as follows:

$$ru(\tau) = \frac{1}{n_e} \sum_{i=1}^{n_e} \sum_{f} ic(f) \cdot \mathbf{1}\left(f \notin P_i(\tau) \wedge f \in T_i\right),$$

$$mi(\tau) = \frac{1}{n_e} \sum_{i=1}^{n_e} \sum_{f} ic(f) \cdot \mathbf{1}\left(f \in P_i(\tau) \wedge f \notin T_i\right),$$

$$S_{\min} = \min_{\tau} \left\{ \sqrt{ru(\tau)^2 + mi(\tau)^2} \right\},$$

where $ic(f)$ is the pre-calculated *information content* of term G_i, given by:

$$ic(G_i) = \log_2 \frac{1}{\Pr(G_i \mid \text{parents of } G_i \text{ in GO})},$$

and $\Pr(G_i \mid \text{parents of } G_i \text{ in GO})$ is the conditional probability of G_i given its parents in the GO structure.

Table S3, S4, S5 reports the performance of GOAnnotator and competing methods in terms of $S_{\min}$. Once again, GOAnnotator achieves a comparbale performance on GOR2023, and outpeforms GORetriever on SP2024 and TR2024 in almost all GO domains, especially on BPO and CCO.

Table S3: Performance comparisons on GOR2023 dataset in terms of $S_{\min}$

Method	MFO	BPO	CCO
**LR-InterPro**	9.762	29.567	8.438
**Net-KNN**	14.506	28.928	7.981
**LR-Text**	11.871	<u>27.612</u>	<u>7.552</u>
**LR-ESM**	9.927	29.917	8.282
**LR-ProtST**	9.646	29.491	8.050
**DeepGOCNN**	14.657	35.235	11.169
**DeepGOPlus**	9.970	29.467	8.392
**Blast-KNN**	9.919	29.786	8.450
**SVM-ESM2**	<u>9.251</u>	29.493	<u>8.002</u>
**GORetriever**	**9.043**	**26.807**	**7.500**
**GOAnnotator**	9.578	27.759	7.701

Table S4: Performance comparisons on SP2024 dataset in terms of $S_{\min}$

Method	MFO	BPO	CCO
**LR-ProtST**	7.307	23.442	6.580
**DeepGOPlus**	<u>6.918</u>	24.441	7.189
**Blast-KNN**	7.096	24.155	6.933
**SVM-ESM2**	**6.899**	23.506	6.475
**GORetriever**	7.173	**22.574**	<u>6.261</u>
**GOAnnotator**	7.162	<u>22.585</u>	**6.177**

Table S5: Performance comparisons on TR2024 dataset in terms of $S_{\min}$

Method	MFO	BPO	CCO
**LR-ProtST**	**7.625**	**19.387**	8.466
**DeepGOPlus**	7.957	29.429	8.109
**Blast-KNN**	8.260	20.032	8.101
**SVM-ESM2**	<u>7.807</u>	<u>19.519</u>	8.173
**GORetriever**	11.330	20.602	8.788
**GOAnnotator**	11.024	20.196	<u>7.825</u>
**Consesus**	7.801	19.517	**7.629**
**Consesus w/o GOAnnotator**	7.448	19.492	8.106

7 Robustness Analysis of GORetriever and GORetriever+

To evaluate the robustness and effectiveness of GORetriever+, we conducted a comprehensive comparison with GORetriever using literature annotation in both Swiss-Prot and TrEMBL, as detailed in Table S6. GORetriever+ outperforms GORetriever at this setting, indicating that GORetriever+ maintains a stronger performance when processing literature that is inconsistent with the distribution of annotated

literature in Swiss-Prot. In real world scenario, this is more practical for the proteins without expert annotated literature.

Table S6: Robustness Analysis of GORetriever and GORetriever+. The test set is a subset of GOR2023 that has been found annotated literature in both Swiss-Prot and TrEMBL.

	MFO	BPO	CCO	Avg.
GORetriever	0.632	0.476	0.593	0.567
GORetriever+	**0.633**	**0.504**	**0.614**	**0.584**

8 Performance Comparisons on SP2024-PT

Table S7: Performance comparison of competing methods on SP2024-PT. The highest values are bolded and the second highest values are underlined. Numbers in parentheses denote the number of test proteins.

Method	MFO (99)	BPO (193)	CCO (244)	Ave. wF_{max}
LR-ProtST	0.664	0.286	0.535	0.495
DeepGOPlus	<u>0.687</u>	0.288	0.518	0.498
BLAST-KNN	0.670	0.263	0.500	0.478
SVM-ESM2	**0.689**	0.297	0.548	0.511
GORetriever	0.661	0.324	0.537	0.507
GOAnnotator	0.660	**0.348**	**0.566**	**0.525**
GOAnnotator_PubTator	0.660	<u>0.339</u>	<u>0.556</u>	<u>0.518</u>

As shown in Table S7, we evaluate the performance of GOAnnotator and competiting methods on SP2024-PT, which is a subset of GOR2023 proteins for which documents could be retrieved using PubTator. The results show: 1) GOAnnotator achieves the best performance of Avg.wFmax, which demonstrates its ability to predict function by annotating relevant literature. 2) GOAnnotator and Pubtator find more relevant literature compare to Siwss-Prot expert's primary annotation. This is because automated tools can comprehensively scan the full database and retrieve the most relevant documents through advanced retrieval and comparison techniques.

9 Performance Comparisons on SP2025

To provide a more comprehensive evaluation, we expanded our analysis to include SP2025, a larger dataset comprising all Swiss-Prot proteins that received their first experimental annotations between January 2024 and March 2025. The results, presented in Table S8, reveal three key findings: 1) GOAnnotator demonstrates superior overall performance compared to GORetriever, particularly in BPO and CCO. This improvement highlights GOAnnotator's ability to efficiently identify and utilize more informative documents through its effective mining approach. 2) Consistent with the SP2024 results, GOAnnotator maintains its best performance in BPO compared to sequence-based methods, further validating its effectiveness in this domain. 3) We observed a slight performance decline in CCO for proteins with post-2024 functional annotations. This observation aligns with our previous findings and likely reflects the incomplete nature of functional annotations for novel proteins.

Table S8: Performance comparison of competing methods on SP2025.

Method	MFO (238)	BPO (519)	CCO (508)	Ave. wF_{max}
LR-ProtST	<u>0.627</u>	0.316	<u>0.547</u>	<u>0.497</u>
DeepGOPlus	0.624	0.306	0.518	0.483
BLAST-KNN	0.625	0.279	0.493	0.466
SVM-ESM2	**0.634**	0.312	**0.552**	**0.499**
GORetriever	0.613	<u>0.334</u>	0.523	0.490
GOAnnotator	0.609	**0.338**	0.532	0.493
GOAnnotator_PubTator	0.465	0.246	0.420	0.377

10 Overlap analysis of GOAnnotator, SVM-ESM2 and BLAST-KNN

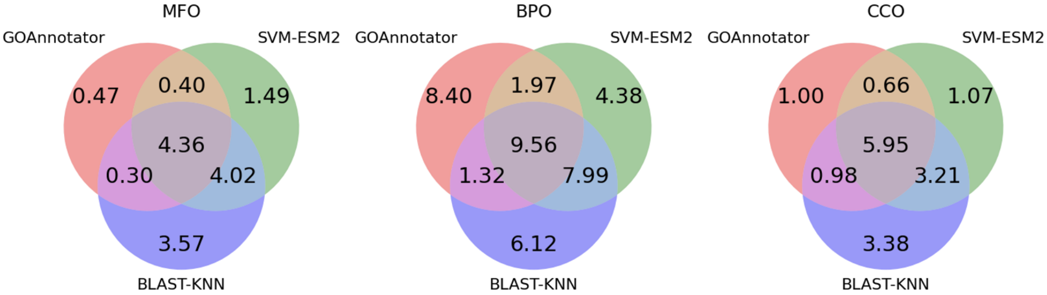

Figure S1: Overlap analysis of GOAnnotator, SVM-ESM2, and BLAST-KNN on TR2024. The numbers indicate the average number of predicted true positive (TP) GO terms per protein for each method.

As shown in Fig. S1, we analyze the overlap and differences among GOAnnotator, SVM-ESM2, and BLAST-KNN on TR2024. Similar to the comparison between GOAnnotator, GORetriever, and GOAnnotator_PubTator, GOAnnotator annotates a vast majority of unique GO terms, especially on BPO and CCO, demonstrating its potential for integration with other methods to achieve the best performance.

11 Case Study

References

[1] Clark, W. T. and Radivojac, P. (2013). Information-theoretic evaluation of predicted ontological annotations. *Bioinformatics*, 29(13):i53–i61.

Table S9: Two Proteins from TR2024 and associated GO terms in CCO, GO definition and informative sentences extracted by GORetriever and GOAnotator.

Protein&GO Term	Sentence from GORetriever	Sentence from GOAnnotator
**PbAQP** (A0A509APT1) **plasma membrane** (GO:0005886)	As the parasites progress to late hepatic stages, PbAQP transcription increases and **PbAQP localizes to the plasma membrane** of hepatic merozoites. (PMID: 32457897)	Either **PbAQP** or PfAQP is a multifunctional channel protein **in the plasma membrane** of the rodent/human malarial parasite for homeostasis of water. (PMID: 29330527)
**Gamma-tubulin** (A0A644F0Y1) **centrosome** (GO:0005813)	**Gamma-tubulin** label was associated with ***perikinetosomal areas of the ventral*** and posterolateral pairs of flagella which are formed de novo during cell division. (PMID: 10928459)	The **gamma-tubulin** complex is a large multiprotein complex that is required for microtubule nucleation at the **centrosome**. (PMID: 11694571)
